# Supplementary material for: Tracking human skill learning with a hierarchical Bayesian sequence model
Source: PLoS Comput Biol. 2022 Nov 30;18(11):e1009866. doi: 10.1371/journal.pcbi.1009866 (PMC9744313; doi:10.1371/journal.pcbi.1009866)
Supplement: S1 Appendix — (PDF) [file pcbi.1009866.s001.pdf]

S1 Appendix

S1.A Algorithm. Parsing the sequence with the ddHCRP as both a generative and recognition model. We use the ddHCRP back-off procedure to obtain the probability of a customer corresponding to the key press, and to update the ddHCRP seating arrangements probabilistically with a new customer corresponding to the actual event (i.e. the instruction that differs from the key press in the case of errors).

$\mathbf{e}$: vector of all events
$\mathbf{k}$: vector of all key presses
$\mathbf{c}$: vector of customer seating arrangement
$\boldsymbol{\alpha}$: vector of HCRP strength values
$\boldsymbol{\lambda}$: vector of HCRP decay rate values
N: maximum context depth

procedure PARSE($\mathbf{e}, \mathbf{k}, \mathbf{c}, \boldsymbol{\alpha}, \boldsymbol{\lambda}$)
    $\mathbf{P} \leftarrow \langle\rangle$
    $t \leftarrow 0$
    while $t < |\mathbf{e}|$ **do**
        if t=0 **then**
            $\mathbf{u} \leftarrow \langle\rangle$
        else
            $left \leftarrow max(0, t-N)$
            $right \leftarrow t-1$
            $\mathbf{u} \leftarrow \mathbf{e}_{left:right}$
        end if
        $\mathbf{P} \frown \langle$ CUSTOMERPROB($\mathbf{u}, k_t, t, \mathbf{c}, \boldsymbol{\alpha}, \boldsymbol{\lambda}$) $\rangle$ ▷ append key press prob. computed by S1.B Algorithm.
        $\mathbf{c} \leftarrow$ADDCUSTOMER($\mathbf{u}, e_t, t, \mathbf{c}, \boldsymbol{\alpha}, \boldsymbol{\lambda}$) ▷ update seating arrangement by S1.C Algorithm.
        $t \leftarrow t+1$
    end while
    return P
end procedure

S1.B Algorithm. Generative process of the ddHCRP: computing the probability of an observation via the back-off procedure.

$\mathbf{u}$: vector of previous events (context)
l: label of the observation
L: number of all labels
t: trial
$\mathbf{c}$: vector of customer seating arrangement
$\boldsymbol{\alpha}$: vector of HCRP strength values
$\boldsymbol{\lambda}$: vector of HCRP decay rate values
n: HCRP level

```
function CustomerProb(u, l, t, c, α, λ, n=None)
    if n=None then
        n ← |u| + 1                              ▷ start back-off procedure from level n
    end if
    if n=0 then
        return 1/L                               ▷ uninformed base distribution
    else
        C_ul ← |c_ul|                            ▷ number of customers with dish l in restaurant u
        old ← Σ_{i=0}^{C_ul} e^{-δc_uli / λ_|u|}  ▷ weighted sum of recency of customers with dish l in restaurant u
        new ← α_|u|                              ▷ prior strength of level
        norm ← α_|u| + Σ_{i=0}^{C_ul} e^{-δc_ui / λ_|u|}
        return old/norm + new/norm × CustomerProb(π(u), l, t, c, α, λ, n − 1)   ▷ back off to level below
    end if
end function
```

Where: $C_{\mathbf{u}l} \leftarrow |\mathbf{c}_{\mathbf{u}l}|$; $old \leftarrow \sum_{i=0}^{C_{\mathbf{u}l}} e^{\frac{-\delta c_{\mathbf{u}li}}{\lambda_{|\mathbf{u}|}}}$; $new \leftarrow \alpha_{|\mathbf{u}|}$; $norm \leftarrow \alpha_{|\mathbf{u}|} + \sum_{i=0}^{C_{\mathbf{u}l}} e^{\frac{-\delta c_{\mathbf{u}i}}{\lambda_{|\mathbf{u}|}}}$; return $\frac{old}{norm} + \frac{new}{norm} \times \textsc{CustomerProb}(\pi(\mathbf{u}), l, t, \mathbf{c}, \boldsymbol{\alpha}, \boldsymbol{\lambda}, n-1)$

S1.C Algorithm. Recognition process of the ddHCRP: update the HCRP seating arrangements with new observation.

$\mathbf{u}$: vector of previous events (context)
l: label of the observation
t: trial
$\mathbf{c}$: vector of customer seating arrangement
$\boldsymbol{\alpha}$: vector of HCRP strength values
$\boldsymbol{\lambda}$: vector of HCRP decay rate values
n: HCRP level

procedure AddCustomer($\mathbf{u}, l, t, \mathbf{c}, \boldsymbol{\alpha}, \boldsymbol{\lambda}$, n=None)
  if n=None **then**
    $n \leftarrow |\mathbf{u}| + 1$ ▷ start back-off procedure from level n
  end if
  if n=0 **then**
    return c ▷ backed off to level 0
  else
    $C_{\mathbf{u}l} \leftarrow |\mathbf{c}_{\mathbf{u}l}|$ ▷ number of previous customers with dish l in restaurant $\mathbf{u}$
    $\mathbf{c}_{\mathbf{u}l} \frown \langle t \rangle$ ▷ append customer timestamp to vector of customers with dish l in restaurant $\mathbf{u}$

    With probabilities proportional to:
    $\sum_{i=0}^{C_{\mathbf{u}l}} e^{\frac{-\delta \mathbf{c}_{\mathbf{u}li}}{\lambda_{|\mathbf{u}|}}}$: ▷ weighted sum of recency of *previous* customers with dish l in restaurant $\mathbf{u}$
    return c ▷ terminate back-off

    $\alpha_{|\mathbf{u}|} \times$ CustomerProb($\pi(\mathbf{u}), l, t, \mathbf{c}, \boldsymbol{\alpha}, \boldsymbol{\lambda}, n-1$): ▷ weighted label probability on level below
    return AddCustomer($\pi(\mathbf{u}), l, t, \mathbf{c}, \boldsymbol{\alpha}, \boldsymbol{\lambda}, n-1$) ▷ back off to level below
  end if
end procedure
