# Supplementary material for: Tracking human skill learning with a hierarchical Bayesian sequence model
Source: PLoS Comput Biol. 2022 Nov 30;18(11):e1009866. doi: 10.1371/journal.pcbi.1009866 (PMC9744313; doi:10.1371/journal.pcbi.1009866)
Supplement: S2 Appendix — (PDF) [file pcbi.1009866.s002.pdf]

S2 Appendix

S2.A Text

We judged that five ddHCRP samples yielded a low-variance estimate of the mean predicted probability of the sequence elements while being computationally sparing (Fig A in S2 Appendix, left). In the case of five samples, the highest coefficient of variation on an example participant's data was below 10% and the median was 3% (Fig A in S2 Appendix, right).

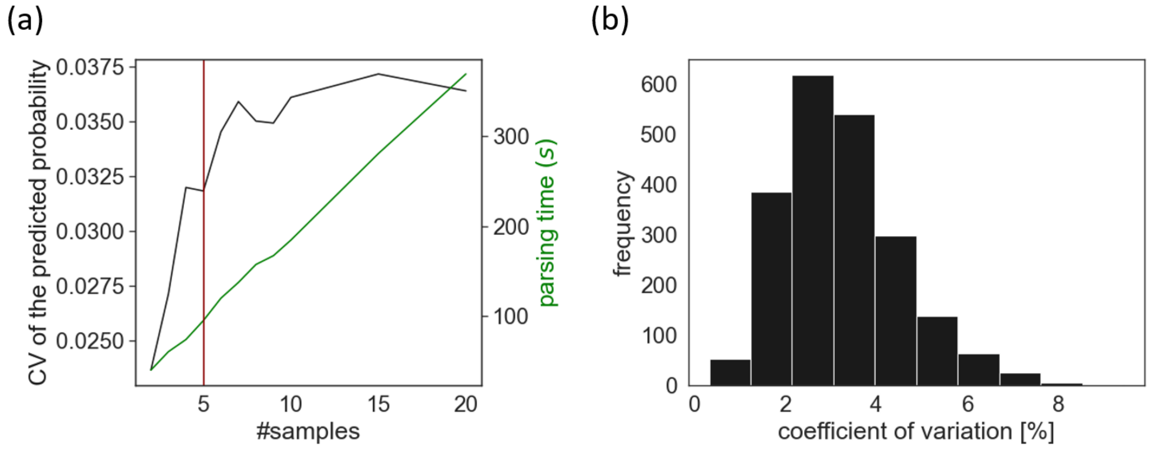

S2.A Fig: (Left) Standard error of the mean predictive probabilities and time required to infer the eating arrangements for different number of ddHCRP samples on the whole data of an example participant. The red line indicates the number of samples used in the Manuscript. (Right) Distribution of the coefficient of variation between five samples for the example data shown in (Left).

Qualitatively, the predictive probabilities appeared unequivocal among the five samples, as exemplified in Fig B in S2 Appendix. We note that the hierarchical structure of our model contributes to the stability of its predictions. Even though there were only five parallel samples, each of them were used to perform smoothing in a robust, hierarchical fashion.

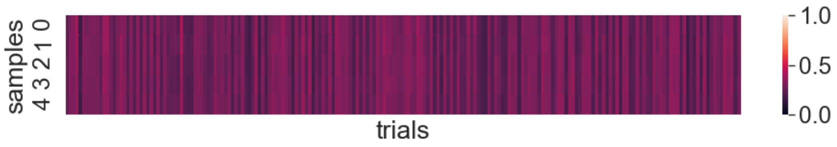

S2.B Fig: Predictive probabilities from the five ddHCRP samples on an example participant's last 200 trials in the last training session.

The response probabilities generated by the model were reproducible across runs of the 1000-iteration random search as well. The median difference between the predictive probabilities generated from two models optimised in two different runs of the random search was .019 (Fig C in

S2 Appendix).This would correspond to a median coefficient of variation of 3.34% (not to be interpreted in the current case of two runs).

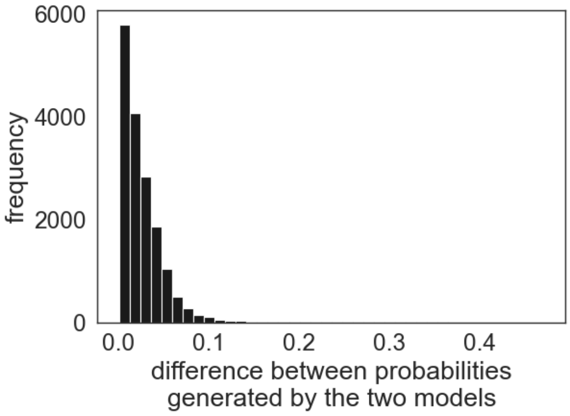

S2.C Fig: Distribution of the difference between the predictive probabilities generated by two models fitted in parallel runs of the random search optimisation to all trials of an example subject.

S2.B Text

Though the learned parameters of the sequence model are directly related to participants' erroneous responses (Figure 10 of the Manuscript), this was not true of the hyperparameters. We computed the proportion of each error type for each participant and session. We assessed the linear relationship between each of the hyperparameters and the proportion of each error type, while controlling for the effect of session. Session was a significant predictor of the proportions of all three error types (all ps < .05) because the proportion of pattern errors gradually increased due to learning, while the proportions of recency errors and other errors reduced (Figure 9 of the Manuscript). This is evidently a behavioral trend that is coherent with the shift in the inferred HCRP hyperparameter values (shown in Figure 4b of the Manuscript). However, the correlations between hyperparameter values and the proportions of either error types were not significant (all ps > .05).

In a similar vein, we analysed the relationship between the inferred hyperparameter values and the relative speeding of errors of different types. There was no significant effect of any hyperparameter on the relative speeding of any error type (all ps > .05). This is not surprising given that the hyperparameters, as discussed in our response to the previous comment, are related only indirectly to the responses. Moreover, the error rate is low in this task ($\sim$10% on average), and subtle effects might not be identified in this small subset of the data. Future error analyses should be carried out in studies employing sequence prediction paradigms, where participants indicate their prediction for the upcoming element rather than reacting to it.

S2.C Text

We compared our main model, the distance-dependent hierarchical Chinese restaurant process (ddHCRP) model, to simpler alternatives. These are inspired by classical *n*-gram learning solutions and can be viewed as ablated versions of the ddHCRP. To ensure meaningful comparisons, all models are based on the distance dependent Chinese restaurant process (ddCRP), that is, they can express priors over the importance of the *n*-grams and exhibit forgetfulness.

Our first, baseline model assumes that participants only learn 2nd-order dependencies, that is, trigrams, and ignore the bigram statistics:

$$p(k_t|e_{t-2:t}) \sim ddCRP(\alpha, \lambda) \tag{1}$$

where k_t denotes the key press at time t, $e_{t-2:t}$ denotes the context of two previous events, and α and λ are the strength and forgetting parameters, respectively. We refer to this model as the Trigram model.

A second model assumes that participants learn a total of N, n-gram levels, independently. Then, for prediction, it assumes that they interpolate the predictive probabilities uniformly across levels:

$$p(k_t|e_{t-N:t}) = \sum_{n=0}^{N} \frac{1}{N} p(k_t|e_{t-n:t}) \tag{2}$$

where each level is an independent ddCRP:

$$p(k_t|e_{t-n:t}) \sim ddCRP(\alpha, \lambda) \tag{3}$$

In this model, the strength and decay rate are equal across levels (otherwise non-equal parameter values across the levels would, in essence, implement weighted interpolation). Since *n*-grams of increasing sizes are learned in parallel by this model, but no weighting is applied to the levels, we refer to it as the uniformly interpolated ddCRP (ddUCRP). The ddUCRP can be viewed as an ablated version of the ddHCRP that performs smoothing without a back-off procedure that would induce preference for levels with more evidence. It is mechanistically simpler than the HCRP and has fewer parameters.

A third model assumes that participants commit to those *n*-gram levels that have accumulated substantial observations. It then does not perform smoothing (this is inspired by the Katz back-off; Katz, 1987):

$$p(k_t|e_{t-N:t}) = \begin{cases} p(k_t|e_{t-n:t}), & \text{if } C > 1 \\ p(k_t|e_{t-n+1:t}), & \text{otherwise} \end{cases} \tag{4}$$

where each level is an independent ddCRP, as in Equation 3.

This model, just like the ddUCRP, tracks a hierarchy of *n*-grams but it does not *use* all the information in a hierarchical manner for prediction. Rather, it uses deterministic back-off in a stack of ddCRPs – thus we call it the stacked ddCRP (ddSCRP). This model can be viewed as another ablated version of the ddHCRP that performs back-off without smoothing. It is *mechanistically* simpler than the HCRP, but has the same number of parameters.

For brevity, we drop the 'dd' from the acronyms and refer to the models as UCRP, SCRP, and HCRP, from here on.

These various models differ particularly in their use of predictive information from deepening windows (corresponding to *n*-grams of increasing *n*) (Table A in S2 Appendix).We therefore considered which aspect of the human behaviour might be most revealing of the differences.

A particularly salient difference between the models is their rendition of smoothing across levels, so we considered the circumstance we expected to show this most clearly - namely the *combination* of predictive information across bigrams and trigrams. That is, consider a 'yellow' trial that was preceded by 'red-blue'. We might expect the 'yellow' response to be hastened by a recent matching trigram trial 'red-blue-yellow'. The question is the extent to which the 'yellow' response is also hastened by 'X-blue-yellow' trials (where 'X'$\neq$'red'). That is, whether the bigram suffix also has a trigram-independent contribution to prediction.

We computed the linear effect of the recency of the bigram suffix occurring in a previous trigram that is not identical to the current one, on the response time at the current trial, for each participant and epoch/session. In the first session (first five epochs), the smoothing effect was 0.4 ms per trial (Fig D in S2 Appendix, right). This means that if the bigram suffix of the current trial was 20 trials more recent than average, the response was faster by 8 ms, independently of trigram frequency. The influence of the bigrams on the responses dropped to a weaker level of 0.15 ms per trial after session 1 and remained stable throughout the training. We refer to this as the smoothing effect.

S2.A Table: Comparison of the model alternatives in terms of statistical features.

	non-parametric	distance-dependence	smoothing	weighting
Trigram	✓	✓	✗	✗
UCRP	✓	✓	✓	✗
SCRP	✓	✓	✗	✓
HCRP	✓	✓	✓	✓

We quantified the smoothing effect exhibited by the four alternative models. All models were fit to participants' responses, while controlling for low-level effects, as described in the Methods of the Manuscript. It is apparent that the trigram model and the SCRP do not capture smoothing behavior. This is because the former does not contain bigram information, the latter soon commits to the trigram information and ignores the bigrams. The UCRP, as well as the HCRP capture the temporal dynamics of the smoothing effect correctly.

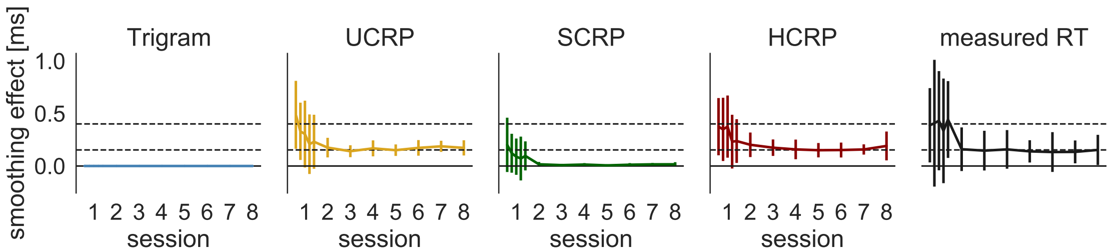

S2.D Fig: The bigram-trigram smoothing effect on the predictions generated by the four alternative models and on participants' measured response times. The dashed lines mark the smoothing effect measured in on participants' responses in the first and last sessions, respectively.

Even though the UCRP model captured the smoothing behavior, by virtue of fixed interpolation, it did so at the cost of underestimating the trigram effect (Fig E in S2 Appendix). While the average measured trigram effect across sessions was 20 ms, the UCRP underestimates it to only 10 ms, while the HCRP commits a milder underestimation of 15 ms.

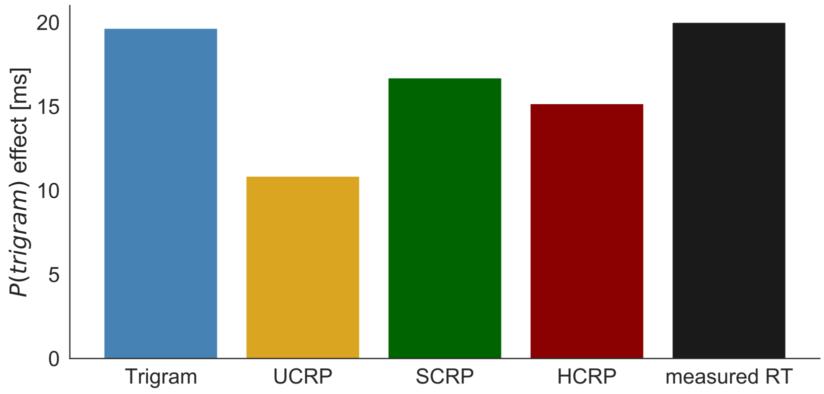

S2.E Fig: Trigram effect on the predictions generated by the four alternative models and on participants' measured response times.

We then examined the failings of the SCRP and UCRP models in more depth – looking across all possible parameter settings in a grid, rather than just the settings that optimized overall model fit. Thus, the SCRP is in fact able to mimic smoothing behavior if it has strong enough forgetting because it alternates in committing to bigrams and trigrams, resulting in an overall influence of the bigrams on the trigrams. However, such smoothing mimicry comes at the cost of stable trigram knowledge, which is why this solution does not emerge in Fig D in S2 Appendix .

Fig F in S2 Appendix shows this dilemma by exhibiting smoothing and trigram effects for the SCRP across parameter settings, and those for the HCRP and the human participants.In the first training session, the SCRP can only emulate the strong smoothing effect exhibited by participants in a very forgetful regime, essentially giving up trigram knowledge (Fig F in S2 Appendix, left). By comparison, in the last training session (Fig F in S2 Appendix, right), the UCRP can not account for the reduction in the smoothing effect while preserving trigram knowledge. The uniform interpolation does not allow for the preferential weighting of the trigrams exhibited by the human participants.

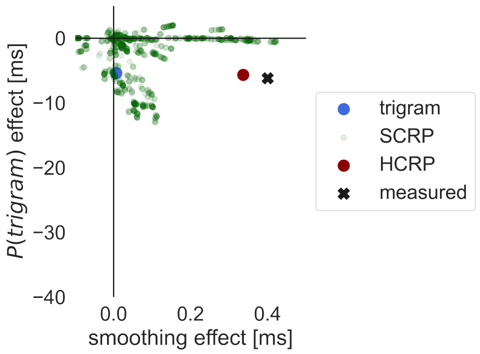

(a) Simulated SCRP effects (in session 1)

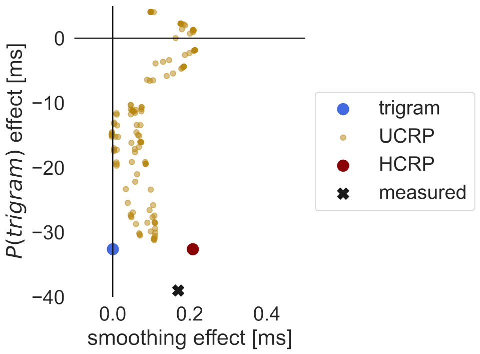

(b) Simulated UCRP effects (in session 8)

S2.F Fig: Comparison of SCRP to HCRP on the first training session and comparison of UCRP to HCRP on the last training session. Each point corresponds to a hyperparameter setting. In the case of the Trigram and HCRP, we used the best fitting hyperparameter settings, as they serve as a reference. The measured effects are averaged across participants.
