# Supplementary figures and images for: Tracking human skill learning with a hierarchical Bayesian sequence model
Source: PLoS Comput Biol. 2022 Nov 30;18(11):e1009866. doi: 10.1371/journal.pcbi.1009866 (PMC9744313; doi:10.1371/journal.pcbi.1009866)

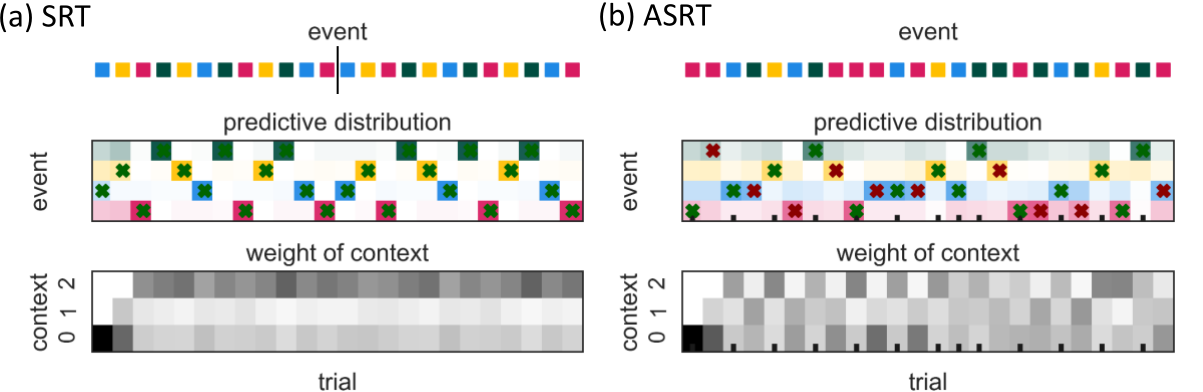

Supplement: S1 Fig — The models were trained with batch learning in order to clearly show how the pattern of predictions depends on the sequence structure without online updates of the model parameters. In (a), the sequence was the concatenation of repeats of a 12-element determinstic pattern (Serial Reaction Time Task or SRT). In (b), the sequence was generated from the ASRT. (Top) Colors denote the sequence elements. The vertical bar marks the boundary between the two repeats in the SRT example segment. (Middle) Predictive probabilities of the four events are shown for each trial. The cells’ hue indicate the event identity, saturation indicates probability value. The Xs indicate the event with the highest predicted probability, i.e. the predicted event; Xs are green for correct predictions and red for incorrect predictions. The ticks at the bottom in (b) indicate high-probability trigram trials. Note that, after having a context of at least two previous elements, all predictions are correct in the case of the deterministic SRT. In the ASRT, incorrect predictions occur for the low probability trigrams. (Bottom) We show what proportion of the predictive probability comes from each context length. Higher saturation indicates a larger weight for a context length. Note that the context of two previous elements is invariably dominant in the SRT predictions where every event is predictable from the previous two. In the ASRT, the context weights follow the largely alternating pattern of the high and low probability trigrams, the former ones being predictable from two previous events, the latter ones being unpredictable. (TIFF) [file pcbi.1009866.s003.tiff]

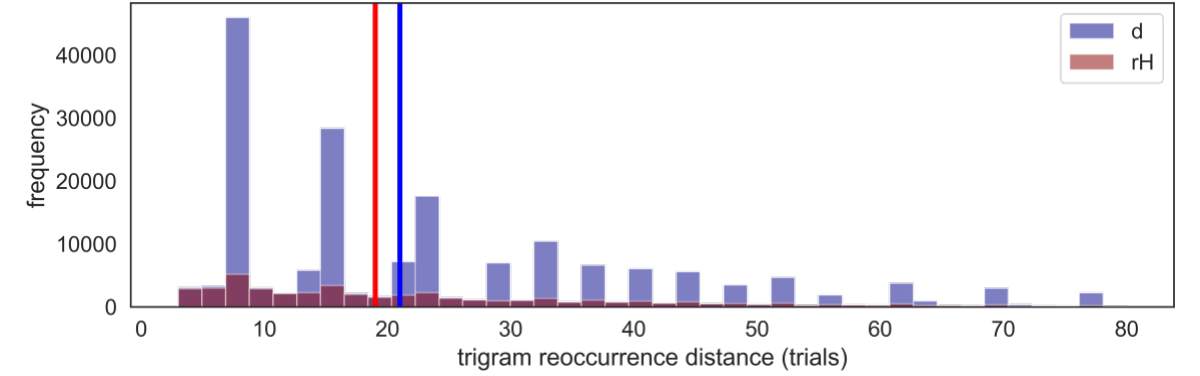

Supplement: S3 Fig — Vertical lines mark the medians. Note the marked periodicity in the case of d trials that imposes a spacing among the trigrams and increases the median reoccurrence distance. (TIFF) [file pcbi.1009866.s005.tiff]

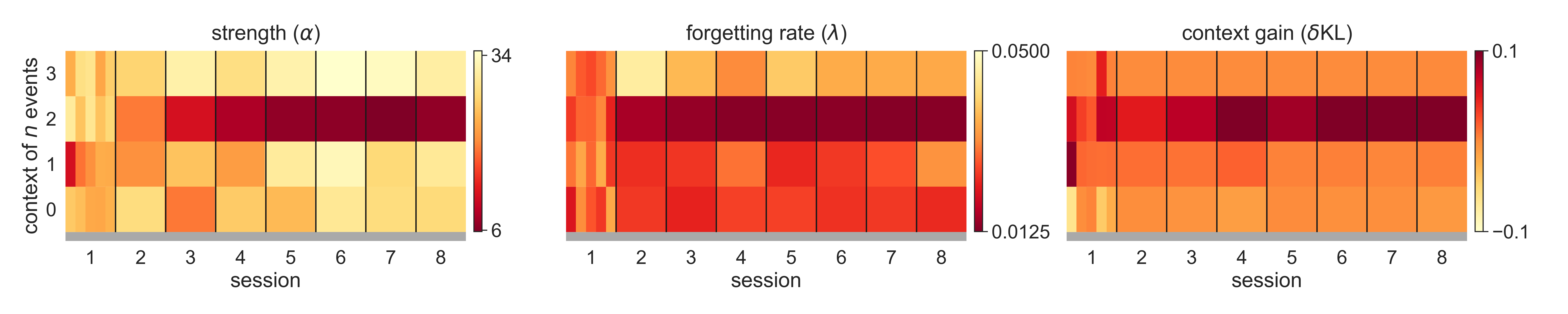

Supplement: S4 Fig — A context of n previous events corresponds to level n in the HCRP. Lower values of α and λ imply a greater contribution from the context to the prediction of behavior. The context gain for context length n is the decrease in the KL divergence between the predictive distribution of the complete model and a partial model upon considering n previous elements, compared to considering only n-1 previous elements. Note that the scale of the context gain is reversed and higher values signify more gain. (TIFF) [file pcbi.1009866.s006.tiff]
