# Supplementary material for: Tracking human skill learning with a hierarchical Bayesian sequence model
Source: PLoS Comput Biol. 2022 Nov 30;18(11):e1009866. doi: 10.1371/journal.pcbi.1009866 (PMC9744313; doi:10.1371/journal.pcbi.1009866)
Supplement: S1 Table — In session 1, the prior was uninformed. In all subsequent sessions, the prior was a truncated Gaussian N’ with the mean of MAP value in the previous session, a fixed variance, and the same interval that the uninformed distributions have in session 1. For most of our results, the first, wider of λ prior was used to allow for extreme forgetfulness or unforgetfulness. For the prediction of response times of errors, we restricted our model to a more forgetful regime by narrowing the λ prior. (PDF) [file pcbi.1009866.s007.pdf]

Table S1: **Hyperparameter prior sets for fitting the response times of all responses (Sections 3.2-.3.6) and errors only (Section 3.7).** In session 1, the prior was uninformed. In all subsequent sessions, the prior was a truncated Gaussian $\mathcal{N}'$ with the mean of MAP value in the previous session, a fixed variance, and the same interval that the uninformed distributions have in session 1. For most of our results, the first, wider of $\lambda$ prior was used to allow for extreme forgetfulness or unforgetfulness. For the prediction of response times of errors, we restricted our model to a more forgetful regime by narrowing the $\lambda$ prior.

| | Session 1 | | Sessions 2-10 | |
|---|---|---|---|---|
| | $\alpha$ | $\lambda$ | $\alpha$ | $\lambda$ |
| all RT | $\mathcal{U}(10^{-4}, 10^{2})$ | $\mathcal{U}(5 * 10^{-4}, 1)$ | $\mathcal{N}'(\alpha_{MAP_{prev}}, 10^{2})$ | $\mathcal{N}'(\lambda_{MAP_{prev}}, 2.5 * 10^{-3})$ |
| error RT | $\mathcal{U}(10^{-4}, 10^{2})$ | $\mathcal{U}(1.25 * 10^{-2}, 1)$ | $\mathcal{N}'(\alpha_{MAP_{prev}}, 10^{2})$ | $\mathcal{N}'(\lambda_{MAP_{prev}}, 2.5 * 10^{-3})$ |
