# Supplementary material for: Tracking human skill learning with a hierarchical Bayesian sequence model
Source: PLoS Comput Biol. 2022 Nov 30;18(11):e1009866. doi: 10.1371/journal.pcbi.1009866 (PMC9744313; doi:10.1371/journal.pcbi.1009866)
Supplement: S2 Table — Due to the large data set, all factors are significant. However, we made an arbitrary cut-off at the horizontal line for the low-level effects included in the response model because of the small effect sizes. (PDF) [file pcbi.1009866.s008.pdf]

Table S2: **Mixed effects model with random intercepts for participants and several low-level predictors, sorted by their absolute fitted slope B (in ms).** Due to the large data set, all factors are significant. However, we made an arbitrary cut-off at the horizontal line for the low-level effects included in the response model because of the small effect sizes.

	B	β	p
intercept	301.192	3.864	<.001
repetition	-36.858	0.306	<.001
error	-21.072	0.296	<.001
post-error	4.566	0.285	<.001
spatial distance	2.107	0.140	<.001
left hand	0.893	0.174	<.001
pre-error trial index	-0.705	0.006	<.001
post-error trial index	0.440	0.006	<.001
