# Supplementary material for: Tracking human skill learning with a hierarchical Bayesian sequence model
Source: PLoS Comput Biol. 2022 Nov 30;18(11):e1009866. doi: 10.1371/journal.pcbi.1009866 (PMC9744313; doi:10.1371/journal.pcbi.1009866)
Supplement: Attachment — Submitted filename: response_letter_1.pdf [file pcbi.1009866.s009.pdf]

We thank the editor and the reviewers for taking the time to read our paper, and for providing invaluable constructive feedback and suggestions. We have addressed all the points, and think that this has greatly improved the Manuscript.

We provide a narrative response to each reviewer's concerns below. The reviewers' comments are color-coded black and the authors' comments are green. Our modifications in the Manuscript are highlighted in red. The modified segments of the Manuscript that were copied in the response letter are indented. The copied segments are identical between the Manuscript and the responses except for the numbering and referencing of the figures, for the sake of readability.

Reviewer #1: This article proposes a novel computational model of sequence learning, and tests some predictions of the model using behavioural data acquired in a variant of the Alternating Serial Response Time (ASRT) task. On each trial, one out of four possible cues is shown and subjects (n=25) have to respond as quickly as possible by pressing a (cue-specific) key. Unbeknownst to the participants, half the cues follow a deterministic second-order sequence, such that the cue at trial t is exactly predictable from the cue at trial t-2. The other half (interleaved with the predictable cues) is random. Participants completed 8 weekly sessions of 2125 trials each (!) where the underlying deterministic sequence was kept fixed, plus 2 additional sessions that include covert changes in the deterministic sequence. Peoples' implicit learning in the task was monitored using response time, which essentially increase when they are surprised. The main result is twofold: (i) after learning, peoples' RT is quicker for predictable cues than for random cues, and (ii) they are also quicker for those random cues that conform (by chance) with the deterministic 2nd-order sequence than for the other random cues. Taken together, these results imply that participants have learned (some aspect of) the task contingencies, albeit entirely implicitly.

Overall, I found the paper interesting and timely. The modelling aspect of the work is novel and promising, and the methods consists of a balanced mixture of computational and experimental approaches, which I feel empathetic with.

Thank you for this positive evaluation. And thank you for the helpful suggestions and criticisms.

Having said this, there are a number of issues that, to my opinion, slightly compromise the quality of the paper (see below). I believe that, if these are addressed in the revised manuscript, this paper would make a significant contribution to the field. Let me now expose the two main concerns I have with the current version of the manuscript.

First of all, I felt that the experimental paradigm was not perfectly appropriate for testing the model. This is essentially because the model is about the ability to flexibility adapt the sophistication of sequence learning to the (hidden) complexity of the sequence. In brief, the model starts with the premise the brain should be equipped with some mechanism that enables it to gradually change its representational dynamics, sic : "We hypothesized that humans build their internal sequence representation in a similar way, starting with learning short-range

dependencies and gradually adding long-range dependencies if they are indeed significantly present in the data" (l. 164-166, p. 6). Accordingly, the model focuses on an optimal (Bayesian) mechanism that can learn any dependency structure (this is the computational problem that the model is meant to solve). However, the dependency structure of the ASRT task is invariant. In other terms, one can solve the task without being able to flexibly adapt to the dependency structure. I would even argue that the main results would be expected, under any form of sequence learning that would capture 2nd-order dependencies. I note that this most likely extends to most (if not all) results reported in the manuscript as it stands. For example, a simple online regression based upon, e.g., truncated Volterra series would make qualitatively identical predictions. Now, I'm not saying people are doing this. Rather, I'm challenging the implicit assumption that the current experimental design offers direct empirical evidence for the model that authors have proposed here. In my opinion, this implies that authors should provide comparative evidence for their model. In other terms, they should try other (simpler?) models, and show that they are less likely explanations for peoples' behavior than the "distance-dependent Hierarchical Chinese Restaurant Process" (hereafter: ddHCRP) model. Importantly, the comparison should be fair, in that candidate models should be a priori able to learn the 2nd-order sequence structure.

We thank the reviewer for the insightful comments and for motivating both the clarification and further justification of our model. We agree that many characteristics of our, fairly complex, distance-dependent Hierarchical Chinese Restaurant Process (ddHCRP) sequence model are not essential for learning stationary 2nd-order dependencies *provided that* it is known a priori that 2nd-order dependencies indeed make up the data. However, our participants were faced with the naturalistic problem of finding dependencies of what to them were unknown orders. Our hypothesis was that individuals have the capacity to solve this unconstrained sequence learning problem with a model that is rich and flexible enough to capture dependencies of various orders, inferring the need for higher orders upon statistical demand. Thus, it would effectively start with a simple model, containing 0th and 1st-order dependencies, and add knowledge of 2nd-order dependencies as the data require this. Crucially, if the learner possesses the machinery for such adaptive complexity, then the *internal* sequence model would be nonstationary in spite of the ground truth being stationary.

In line with this, previous work has shown that learning of 2nd-order dependencies is difficult and requires favorable conditions, for instance, cuing nonadjacent pairs to perceptually enhance their grouping (Creel, Newport, & Aslin, 2004; van den Bos, Christiansen, & Misyak, 2012; Szegedi-Hallgató et al., 2017), increasing the variability of the intervening stimulus (AxB, AyB, AzB…) (Gómez, 2002; Romberg & Saffran, 2013), or extensive training, like in the current paper. In contrast, learning of first-order dependencies emerges faster and is much more robust across participants (e.g. Hunt & Aslin, 2001 and Gómez, 2002). This led us to think that individuals have a prior that emphasizes first-order dependencies, but allows for higher orders if demanded by the task. Rather than measuring individuals' distance to the ground truth with a predefined 2-order sequence model, like a truncated Volterra series with a window containing two previous time steps, we sought to track the process by which they arrive at such a model.

In response to the reviewer's concern, we now compare the ddHCRP to three alternative, simpler higher-order sequence models: a trigram model, a uniform interpolation model, and a backoff model. Each of them possesses a subset of the features of the ddHCRP. As such, the alternative models can be viewed as ablated versions of the ddHCRP. To ensure fair comparisons, all models are based on the distance dependent Chinese restaurant process (ddCRP), that is, they can express priors over the importance of the *n*-grams and exhibit forgetfulness. To highlight the key differences between the models, we assessed them on two essential aspects of the human data: participants' sensitivities to trigram probabilities and their chunk smoothing behavior (the way their reaction times appear to combine trigram and bigram dependencies). In brief, we found that the ablated models could account for either aspect in isolation. However, they could not account for both these effects with a single setting of their parameters. This suggests that the characteristics of the HCRP that implement a weighted chunk smoothing are essential to explain participants' sequential behavior. We included these results in the Supplementary Text S2.C, but, since they are rather extensive, have not copied them into this response. We summarise the results in a short result subsection in the Manuscipt:

> "2.8 Alternative models
>
> The principled back-off mechanism of the HCRP model was essential to account for participants' smoothing behavior – namely, that they flexibly combined higher- and lower-order information for prediction. Ablated alternative models listed in Table S2.A, even if containing multi-order sequence information, fell short in explaining how participants combined the multi-order information locally, while also maintaining stable knowledge of the global chunk statistics (Figure S2.D, Figure S2.E, and Figure S2.F)."

Second, I have a few issues with the presentation of the model, as it stands. In brief, if one is not already cognisant of Dirichlet processes, then one cannot understand how the model works. There are only 3 equations in the manuscript that relate to the model, and they clearly are not sufficient to describe the model. This is PLoS Computational Biology: authors should not be reluctant to be explicit about the mathematics :) As I am sure authors are well aware, the typical way of describing a bayesian learning/inference algorithm is to start with the generative model, and then describe the model inversion procedure. Equations 1-3 are summarizing the main aspect of the generative model, but describing the full generative model requires more details.

We apologise for not providing more information. We now describe the generative and recognition processes of the ddHCRP model in full detail in the second paragraph of Section 1.4 of the Manuscript:

> "As a first step of our ABC procedure, we parsed e and k chronologically, such that the probability of a key press at t was influenced by observations up to t − 1, modeling sequential information accumulation (Algorithm S1.A). At each time step, the HCRP operated as both the generative and recognition model, based on the same hierarchical back-off scheme. On trial t, we evaluated the probability of seating a new customer to a table serving dish kt, according to the generative process (Algorithm S1.B). This corresponded to computing the predictive probability p(k_t) of the participant's response.

> Then, the seating arrangement was updated by seating a customer to a table serving the dish et, according to the recognition process (Algorithm S1.C). This corresponded to updating the participant's internal model with the event et, that is, the required response (which, in the case of erroneous responses, was different from the actual response kt). As such, we modeled the generative process of the actual responses k as a function of having learnt the required responses e."

Here, we provide pseudocode for readers with a more computational bent. The three algorithms make the mathematical details explicit. Algorithm S1.A describes how the vector of the task instructions and participants' responses was parsed by the HCRP. Algorithm S1.B describes the generative process of the ddHCRP, containing the equations for the recursive computation of the weights of the shallowing contexts. Algorithm S1.C describes the recognition process of the ddHCRP, containing the equations for the probabilistic update rules of the model. We refer the reader to Algorithm S1.C earlier as well, in Section 1.3, when first introducing the HCRP:

> "Here, we have Chinese restaurants on N +1 levels, each level modeling the probability distribution over responses given a context of n previous events. [...] The 'reliance' of deeper contexts on shallower ones is realised by a back-off procedure (Algorithm S1.C)."

We believe that the pseudocode we provided is the most succinct way to ensure the understandability and reproducibility of our model.

More importantly, no computational detail is given regarding model inversion. Does it rely on sampling, or some variational approximation (the latter, I guess, since they cite Beal and colleagues)? The authors should insert a complete model/methods subsection on model inversion, with full details regarding the algorithmic approach. For example: how is it initialized? What summary statistics are used and how are they updated online? How does computational complexity grow (Dirichlet processes rely on some threshold to augment their state-space: what is it here?) ? They should use this description to highlight the core computational properties of the ddHCRP learning algorithm. In relation to the first comment above, this may also serve to motivate the choice of candidate alternative models, and discuss possible pros and cons from a computational perspective.

We apologise for not being clear on model inversion in the first draft. We updated Paragraph 2 of Section 1.4 of the current Manuscript with the aforementioned Supplementary Algorithms that are used to to infer the ddHCRP seating arrangements, that is, the samples of the hierarchical Dirichlet process, given participants' observations.

We used ABC for fitting the hyperparameters of the HCRP. We describe this in the rest of Section 1.4 of the Manuscript (1.4 Parameter fitting):

> "Given the sequence presented to the participants, their responses, and response times, we are interested in finding the parameter values of the low-level effects and the internal sequence model that most likely generated the behavior. We assumed that the likelihood of the log response times was a Gaussian distribution with a mean value of the log

response times predicted by the full model. We performed approximate Bayesian computation (ABC) to approximate the maximum a posteriori values of the parameters of interest:

$$\underset{\boldsymbol{\theta},\boldsymbol{\rho}}{\operatorname{argmax}} P(\boldsymbol{\theta}|\mathbf{e},\mathbf{k},\boldsymbol{\tau},\sigma)$$

where θ is the parameter vector of the HCRP comprising the strength parameters α and forgetting rate parameters λ; ρ is the vector of response parameters, including the weights of the low-level effects, the weight of the HCRP prediction, and the response noise; e is the sequence of events (mapping onto required responses); and k is the sequence of key presses (actual responses); τ is the sequence of response times; and σ is the Gaussian noise of the response time."

We start the ABC procedure with parsing the sequence of participants' observations and responses, given a random initial set of hyperparameters. Concurrently with updating the seating arrangements trial-by-trial, we generate predictions of the upcoming sequence element. This predictive probability was transformed into predicted response times assuming a logarithmic mapping of internal expectations to response times. As described in Paragraph 3 of Section 1.4 of the Manuscript, having generated the response time predictions, we evaluated the likelihood of participants' measured response times assuming Gaussian noise. Finally, Paragraph 4 of Section 1.4 of the Manuscript details the optimisation method, i.e. how we iteratively searched for values of the ddHCRP hyperparameters θ and the response parameters that increased the likelihood of participants' response times.

Regarding computational complexity, the bulk of the computation in the ddHCRP lies in calculating the likelihood ratio of seating customers to old or new tables. As opposed to the traditional CRP where customer identity can be ignored and new seating assignments are guided by summary statistics (i.e. the number of customers at the tables), in the distance-dependent case we need to store the timestamps of all individual customers and compute their recency for new seating assignments. This increases both the memory complexity and the computational complexity of the ddHCRP compared to a traditional HCRP. Since individual data points are used for inference rather than summary statistics, the expected number of customers per restaurant grows linearly with the data (Figure R1).

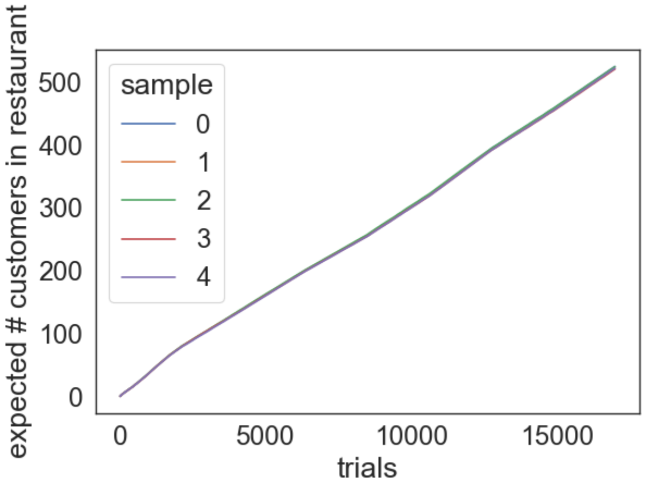

Figure R1. The expected number of customers per restaurant across 1700 data points (all training sessions) of an example participant.

I have other more minor concerns, but I don't believe it is useful to discuss these unless authors are willing to address those two issues first. I hope authors understand my concerns, and take this review round as an opportunity to improve the paper (which I think deserves to be published in PLoS CB, provided the above concerns are adequately addressed)!

We appreciate the opportunity and pointers to improve the paper. We hope that the reviewer will see that we have addressed these concerns with detailed additions to the paper, and look forward to understanding any remaining issues.

Reviewer #2: Learning increasingly complex statistical regularities present in continuous sequences is a difficult problem affected by the curse of dimensionality. Yet, humans can take advantage of such regularities to speed up their responses during perceptuo-motor tasks. In this manuscript, the authors adapt an existing model of language processing to capture the learning process of participants practicing a visuo-motor task over multiple weeks.

Overall, the model elegantly capture many behavioural hallmarks of implicit sequence learning, and thus appear as a promising quantifying tool.

Thank you for this positive assessment. And thank you for the helpful suggestions and criticisms.

The manuscript focuses on validating the predictions of the model, laying the groundwork for future studies in cognitive neurosciences.

I have two major comments and few minor points I would like the authors to address before considering publication.

Major comments:

- I might have missed some supplementary material, but I could not find the formal description of the model. I understand the hierarchical Dirichlet process is relatively standard, but the details of its implementation and in particular all the adaptations of the response mapping (low level biases), the explicit rules for selecting and weighting levels, and the inversion routine must be included, albeit in an annex, both for the sake clarity and to ensure self-sufficiency of the manuscript and therefore reproducibility. The absence of a complete set of equations describing the learning rules and the response function, combined with the lack of typographic distinction between scalar and vector parameters, makes the read rather difficult. The author should also make sure to make their code available in a public repository for completeness.

We apologise for not providing more information. We now describe the generative and recognition processes of the ddHCRP model in full detail in the Paragraph 2 of Section 1.4 of the Manuscript:

> “As a first step of our ABC procedure, we parsed e and k chronologically, such that the probability of a key press at t was influenced by observations up to t − 1, modeling sequential information accumulation (Algorithm S1.A). At each time step, the HCRP operated as both the generative and recognition model, based on the same hierarchical back-off scheme. On trial t, we evaluated the probability of seating a new customer to a table serving dish kt, according to the generative process (Algorithm S1.B). This corresponded to computing the predictive probability p(k_t) of the participant’s response. Then, the seating arrangement was updated by seating a customer to a table serving the dish et, according to the recognition process (Algorithm S1.C). This corresponded to updating the participant’s internal model with the event et, that is, the required response (which, in the case of erroneous responses, was different from the actual response kt).

> As such, we modeled the generative process of the actual responses k as a function of having learnt the required responses e."

Here, we provide pseudocode for readers with a more computational bent. The three algorithms make the mathematical details explicit. Algorithm S1.A describes how the vector of the task instructions and participants' responses was parsed by the HCRP. Algorithm S1.B describes the generative process of the ddHCRP, containing the equations for the recursive computation of the weights of the shallowing contexts. Algorithm S1.C describes the recognition process of the ddHCRP, containing the equations for the probabilistic update rules of the model. We refer the reader to Algorithm S1.C earlier as well, in Section 1.3, when first introducing the HCRP:

> "Here, we have Chinese restaurants on N +1 levels, each level modeling the probability distribution over responses given a context of n previous events. [...] The 'reliance' of deeper contexts on shallower ones is realised by a back-off procedure (Algorithm S1.C)."

We believe that the pseudocode we provided is the most succinct way to ensure the understandability and reproducibility of our model.

We made sure to typeset vector parameters in bold throughout the corrected version of both the Manuscript and the Appendix.

Finally, we have published the data and code on github: https://github.com/noemielteto/HCRP_sequence_learning linked in the Data and code availability section of the Manuscript.

- The model is entirely fitted to the RTs of actual responses, which is perfectly understandable and well justified in the manuscript. However, the structure of the model should allow to also predict the "motor choices", and more interestingly, make non-trivial predictions about errors (eg. generalisation errors). The authors should provide some hints about this could be implemented or, better, provide some additional analyses addressing this point.

Having a look at such qualitative predictions might be particularly critical eg. in section 2.7 in which 'pattern errors' (coming from learned expectations) are opposed to 'recency' and 'other' errors: if the differential in RT between those cases is indeed coming from the expression of some learned >2-order contingencies, choices qualifying as 'pattern errors' should be aligned with the internal 'seating pattern' recovered by the learning model (but not in the case of other errors). Addressing this point would make a fair sanity check of the interpretation of the error speeding in pattern errors.

We are grateful to the reviewer for this astute observation, namely that the speeding of errors should be directly related to the HCRP seating arrangements. That is, the recency of the accumulated observations is related to the likelihood of responding contingent on the context of those earlier observations, which sometimes leads to errors in the task. In fact, the hierarchical nature of our model means that it is the *ratio* of the recency of observations in the current context and a shallower one that determines the response. This ratio corresponds to the seating odds on the current level or a shallower one in the HCRP metaphor. In Fig 6a, bottom and Fig

6b, bottom in the Manuscript, we demonstrated how the seating odds on level 2 alternate depending on the trial type: in high-frequency triplet trials, level 2 in the model is used for prediction, whereas on low-frequency triplet trials it is effectively ignored.

We have included the analysis below, that is exactly in line with the reviewer's expectations, in Section 2.7 of the Results in the Manuscript:

> In order to elucidate the relationship between the inferred seating arrangements in the HCRP_f model and participants' errors, we computed the average seating odds for each level across the three error types (the same measure as in Figure 6a and b, bottom of the Manuscript). As shown in Figure R2 (now also Figure 10 in the manuscript), the weight of level 2 in the HCRP_f, that is, the recency of trigrams, did not influence the speed of different types of errors to the same degree ($F(2, 72) = 39.90$, $p<.001$). The weight of level 2 was stronger in the case of recency errors than other errors ($t = -2.72$, p=.009), and it was even stronger in pattern errors than recency errors ($t = -5.61$, p<.001). The overall difference in the weight of level 0, that is, the recency of unigrams ($F(2, 72) = 20.94$, $p<.001$) was driven by the opposite trend. The weight of the unigram observations was stronger when participants committed other errors than recency errors ($t = 2.35$, $p=.02$), and stronger in the case of recency errors than pattern errors ($t = 3.89$, $p<.001$). There were significant differences among the error types in the weights of level 1 and 3 as well, though more modest and not three-way ($F(2, 72) = 10.26$, $p<.001$; $F(2, 72) = 8.02$, $p<.001$, respectively). Overall, these results clarify the relationship between the learned parameters of the HCRP, i.e. proportions of observations contingent on deepening contexts, and the types of errors that participants made.

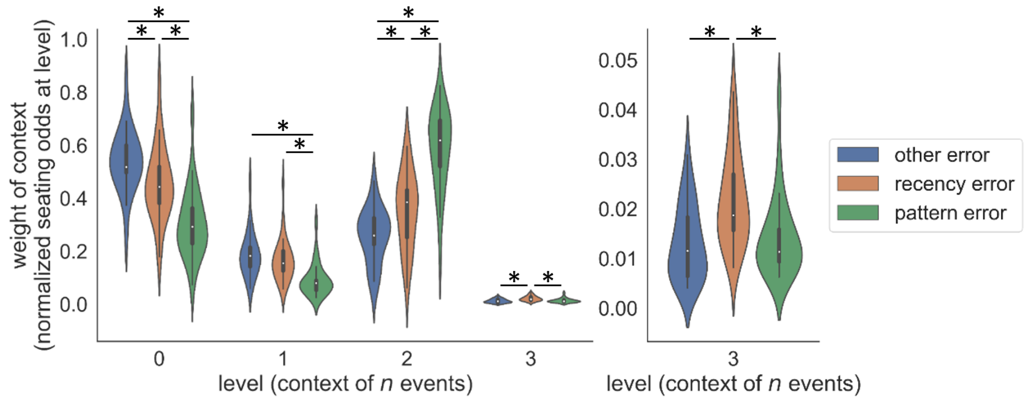

Figure R2. (Left) Weights of the HCRP levels differ across error types. (Right) Zoomed in for HCRP level 3 only.

Another prediction would be that participants/phases characterised by a deeper representation should also exhibit specific types of error reflecting their higher order expectations. I understand such events might be rare and therefore hard to analyse, but they could provide some further insights into the behavioural variability across participants, which is a bit lacking in the current

manuscript, especially for a model intended as a quantification tool for behavioural neurosciences.

We included the response to this question in Supplementary Text S2.B:

> Though the learned parameters of the sequence model are directly related to participants' erroneous responses, as shown above, this was not true of the hyperparameters. We computed the proportion of each error type for each participant and session. We assessed the linear relationship between each of the hyperparameters and the proportion of each error type, while controlling for the effect of *session*. *Session* was a significant predictor of the proportions of all three error types (all *ps* < .05) because the proportion of pattern errors gradually increased due to learning, while the proportions of recency errors and other errors reduced (Figure R3; now Figure 8 in the main Manuscript). This is evidently a behavioral trend that is coherent with the shift in the inferred HCRP hyperparameter values (shown in Figure 4b of the Manuscript). However, the correlations between hyperparameter values and the proportions of either error types were not significant (all *ps* > .05). In a similar vein, we analysed the relationship between the inferred hyperparameter values and the relative speeding of errors of different types. There was no significant effect of any hyperparameter on the relative speeding of any error type (all *ps* > .05). This is not surprising given that the hyperparameters, as discussed in our response to the previous comment, are related only indirectly to the responses. Moreover, as suggested by Reviewer 2, the error rate is low in this task (10% on average), and subtle effects might not be identified in this small subset of the data. Future error analyses should be carried out in studies employing sequence prediction paradigms, where participants indicate their prediction for the upcoming element rather than reacting to it.

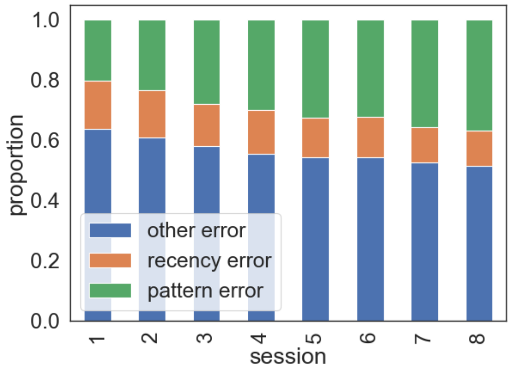

Figure R3. Proportions of errors of different types across sessions.

Minor comments:

- I understand where the Chinese restaurant example comes from, but the back and forth between this terminology and the presented experimental design is a bit hard to follow.

Sentences like 'the customers respond to certain key presses' or 'the probability of sitting at a table ... predict how likely each key is' are a bit nonsensical for a reader not familiar with Chinese restaurant processes. If the author insist on keeping the CRP example, I would suggest doing it in one place, then map each terms to both the equations (cf my first point) and the respective experimental concepts (eg. what is a table in terms of sequence representation?).

We are sorry that our descriptions were confusing. As the reviewer implies, the Chinese restaurant metaphor is pretty well established in conventional (and cognitive science) descriptions of non-parametric Bayesian modeling. However, we have adopted the reviewer's suggestion of being much more explicit about linking its terminology (tables, customers, etc) to the components of the ASRT. In Table 1 of the Manuscript, we included a CRP-to-experiment terminology mapping. We hope that this now makes our intent clear.

Table 1. Terminology of the hierarchical Chinese restaurant process mapped onto the experimental measures of the current study.

ddHCRP metaphor	Experimental measures
customer	observation at t
table	subset of observations up to $t-1$
dish	label of the observation at t (key press k_t or event e_t)
restaurant on level n	context of n previous events ($\mathbf{e}_{t-n:t-1}$)

- p.10: "We parsed the sequence five times..." Why five? This seems a very low for a sampling procedure. Or is this meant at the trial level, therefore exhausting the possible seating arrangements?

The five samples do not refer to exhausting the possible seating arrangements but rather to repeating the seating decision five times and tracking the seating arrangements in the five parallel samples. We share the Reviewer's concern that five is a low number of samples, in general. We provide an analysis in Supplementary Text S2.B showing that five samples are sufficient in this particular case:

> We judged that five ddHCRP samples yielded a low-variance estimate of the mean predicted probability of the sequence elements while being computationally sparing (Figure R3, left). In the case of five samples, the highest coefficient of variation on an example participant's data was below 10% and the median was 3% (Figure R3, right).

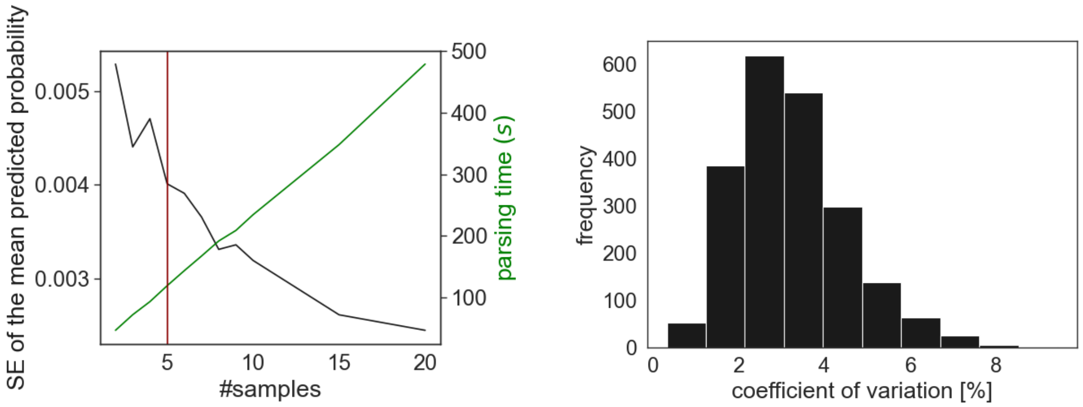

Figure R3. (Left) Standard error of the mean predictive probabilities and time required to infer the eating arrangements for different number of ddHCRP samples on the whole data of an example participant. The red line indicates the number of samples used in the Manuscript. (Right) Distribution of the coefficient of variation of the predictive probabilities between five samples for the example data shown in (Left).

Qualitatively, the predictive probabilities appeared unequivocal among the five samples, as exemplified in Figure R4. We note that the hierarchical structure of our model contributes to the stability of its predictions. Even though there were only five parallel samples, each of them were used to perform smoothing in a robust, hierarchical fashion.

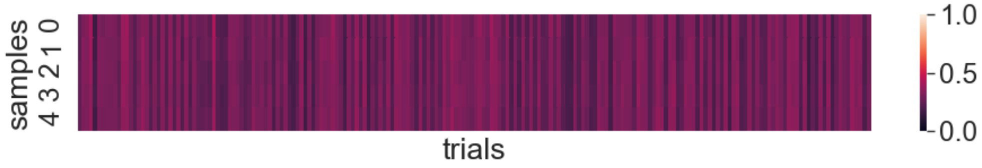

Figure R4. Predictive probabilities from the five ddHCRP samples on an example participants' last 200 trials in the last session.

We refer the reader to to the above analysis in Paragraph 2 of Section 1.4 of the Manuscript:

> "We parsed the sequence five times to generate five seating arrangements. p(kt) was averaged over the five seating arrangement samples (yielding a relatively high-precision estimate of p(kt); Figure S5)."

- On the same topic, it would be helpful to have some measure of convergence of the inversion procedure (eg. variance of the estimates across different runs of the random search)?

We provide an analysis of the inter-run variance in Supplementary Text S2.A:

The response probabilities generated by the model were reproducible across runs of the 1000-iteration random search as well. The median difference between the predictive probabilities generated from two models optimised in two different runs of the random search was .019 (Fig R5). This would correspond to a median coefficient of variation of 3.34% (not to be interpreted in the current case of two runs).

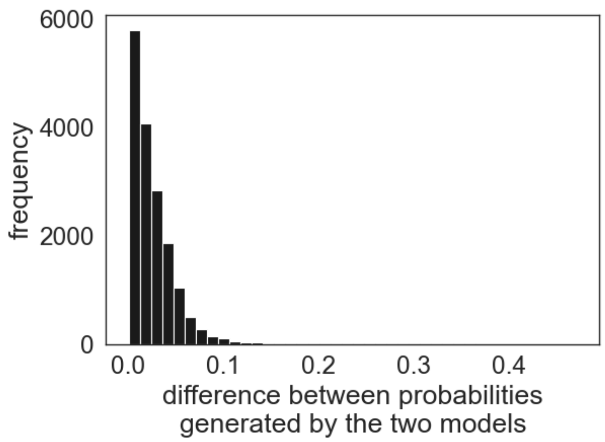

Figure R5. Distribution of the difference between the predictive probabilities generated by two models fitted in parallel runs of the random search optimisation to all trials of an example subject.

We refer the reader to the above analysis in Paragraph 3 of Section 1.4 of the Manuscript:

> "In order to approximate θ and ρ that maximize this likelihood, we performed random search in the space of θ for 1000 iterations (for the convergence of the random search procedure, see Figure S7)."

- Is there a correlation between the lambdas at the different levels? What is the rationale for not having identical values across levels?

There was a significant correlation between λ_1 and λ_2 (r = 0.358, p < .001) and λ_2 and λ_3 (r = 0.269 p < .001). Other pairwise correlations among lambda values were not significant (all *p*s > .001). The Reviewer was right to suspect that not all the hyperparameter values of our model were independent and this reduces the interpretability of the inferred parameter values to some extent. However, parametrizing our model as such was essential for our purposes. The rationale for having different forgetting rates across levels was to be able to capture forgetting that is context-depth dependent. We added a note in Paragraph 4 of the Discussion about these issues:

> "[...] here, skill is associated with more sophisticated methods of managing regular external variability. Simply put, learners can change the size of chunks they choose to remember better. The context-depth specific parametrisation of our model allows for the

identification of other potential practice-related changes, which were actually not observed in the ASRT data, but are often observed in other tasks. For instance, in case of higher memory demands, it is possible that participants improve their performance by shifting the strength of a chunk size required by the task but remain forgetful about chunks of that size. This would result in behavior that is increasingly contingent on the correct chunk size but noisy due to over-reliance on recent observations."

- Table 1: effect size (in ms) should be provided to allow comparison between actual and predicted RT effects.

We have added the unstandardized beta coefficients (in units of ms) to Table 2 (former Table 1) of the Manuscript in order to facilitate the comparisons between predictions and measurements:

Table 2. Repeated measures ANOVAs in sessions 1-8. In the left set of columns, the *trial type* is defined as the state and in the right set of columns it is defined as P(trigram).

	effect	B [ms]	F	p	effect	B [ms]	F	p
measured RT	*session*	-10.11	133.38	<.001	*session*	-9.33	97.81	<.001
	state	-1.99	116.01	<.001	*P(trigram)*	-13.68	272.66	<.001
	*session*state*	-3.63	29.07	<.001	*session*P(trigram)*	-2.72	9.25	<.001
predicted RT	*session*	-11.06	216.68	<.001	*session*	-10.19	203.70	<.001
	state	-3.16	171.44	<.001	*P(trigram)*	-7.02	229.83	<.001
	*session*state*	-2.76	44.92	<.001	*session*P(trigram)*	-3.01	26.44	<.001

Reviewer #3: In “Tracking human skill learning with a hierarchical Bayesian sequence model” Eltetö and colleagues apply a hierarchical sequence model to reaction time data from an alternating serial response time task. The hierarchical Chinese restaurant process (HCRP) with each level of the hierarchy coding for sequences of a particular length is enriched with a process of forgetting (forgetful HCRP). The model is then applied to data from 25 participants who completed 10 runs with a total of more than 20000 trials. The first 8 runs were generated by the same statistical trigram structure, while sessions 9 and 10 where used to probe the stability of the learned sequences against novel trigrams. In combination with some low level feature such as repetition and error-related effects, the model was able to capture how participants learned the sequence. The success fitting is illustrated by the correlation of predicted traces to left out data. The fitted parameters capture the nature of the task (over session) and, importantly, correlate with working memory indices acquired with classical working memory tasks.

This is a very nice application of a model for implicit learning tested on a rich dataset that has enough trials (25 participants, >20000 trials each) to actually allow to fit the model and investigate effects of changing structure in the task. Both task and model are well explained. I have few comments and questions on the model and model fitting to the authors (see below).

Jakob Heinzle

Thank you for the positive opinion and the appreciation of our Manuscript. And thank you for the helpful suggestions and criticisms.

Major:

Model fitting: It was not entirely clear to me how ABC was applied, here.

We apologise for the lack of detail in our fitting procedure. We have updated section 1.4 ‘Parameter fitting’ (lines 297-385) to be precise, and in particular to address your questions.

First, we updated the description of the generative and recognition processes of the HCRP by which we generated the predictions:

> “As a first step of our ABC procedure, we parsed e and k chronologically, such that the probability of a key press at t was influenced by observations up to t − 1, modeling sequential information accumulation (Algorithm 1). At each time step, the HCRP operated as both the generative and recognition model, based on the same hierarchical back-off scheme. On trial t, we evaluated the probability of seating a new customer to a table serving dish kt, according to the generative process (Algorithm 2). This corresponded to computing the predictive probability p(k_t) of the participant’s response. Then, the seating arrangement was updated by seating a customer to a table serving the dish et, according to the recognition process (Algorithm 3). This corresponded to updating the participant’s internal model with the event et, that is, the required response (which, in the case of erroneous responses, was different from the actual response kt).

As such, we modeled the generative process of the actual responses k as a function of having learnt the required responses e.”

Further, we address the particular questions raised:

You describe that you simulated five instances of new seatings in every trial and then averaged over them. Is this enough to get stable fits? How could you assure this, other than by looking at the correlation to the held-out data?

As the Reviewer might imagine, fitting is computationally costly. We chose five samples based on the variability in the estimate of the mean predicted probabilities of the sequence elements. We added our justification to Supplementary Text S2.A, showing that five samples are sufficient in this particular case:

We judged that five ddHCRP samples yielded a low-variance estimate of the mean predicted probability of the sequence elements while being computationally sparing (Figure R6, left). In the case of five samples, the highest coefficient of variation on an example participant’s data was below 10% and the median was 3% (Figure R6, right).

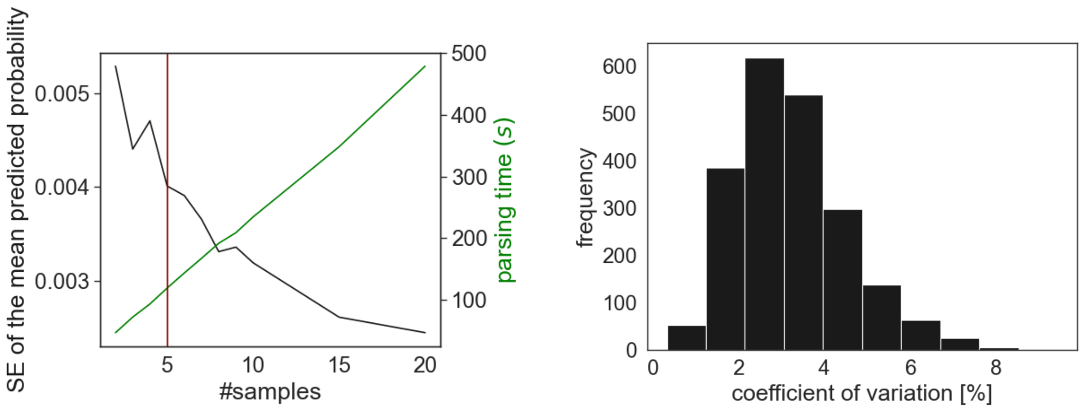

Figure R6. (Left) Standard error of the mean predictive probabilities and time required to infer the eating arrangements for different number of ddHCRP samples on the whole data of an example participant. The red line indicates the number of samples used in the Manuscript. (Right) Distribution of the coefficient of variation of the predictive probabilities between five samples for the example data shown in (Left).

Qualitatively, the predictive probabilities appeared unequivocal among the five samples, as exemplified in Figure R7. We note that the hierarchical structure of our model contributes to the stability of its predictions. Even though there were only five parallel samples, each of them were used to perform smoothing in a robust, hierarchical fashion.

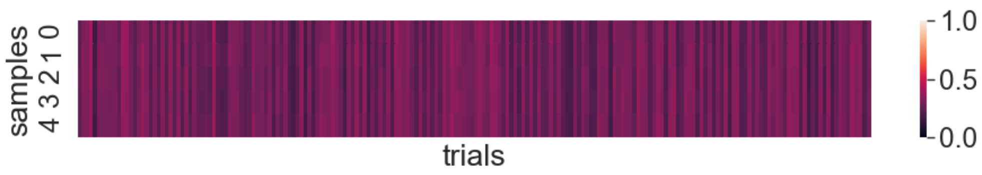

Figure R7. Predictive probabilities from the five ddHCRP samples on an example participants' last 200 trials in the last session.

We refer the reader to to the above analysis in Paragraph 2 of Section 1.4 of the Manuscript:

> "We parsed the sequence five times to generate five seating arrangements. p(kt) was averaged over the five seating arrangement samples (yielding a relatively high-precision estimate of p(kt); Figure S5)."

- On the same topic, it would be helpful to have some measure of convergence of the inversion procedure (eg. variance of the estimates across different runs of the random search)?

We provide an analysis of the inter-run variance in Supplementary Text S2.A:

> The response probabilities generated by the model were reproducible across runs of the 1000-iteration random search as well. The median difference between the predictive probabilities generated from two models optimised in two different runs of the random search was .019 (Fig R8). This would correspond to a median coefficient of variation of 3.34% (not to be interpreted in the current case of two runs).

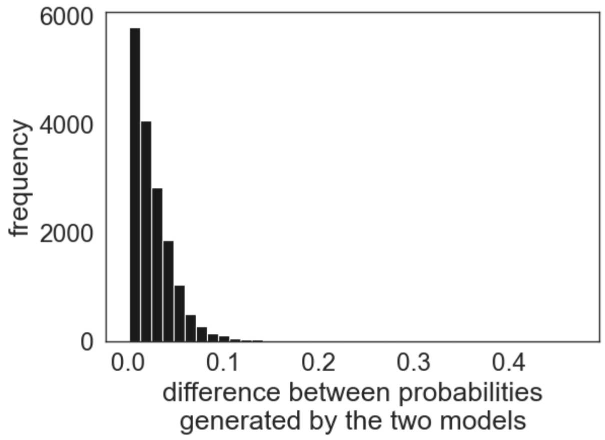

Figure R8. Distribution of the difference between the predictive probabilities generated by two models fitted in parallel runs of the random search optimisation to all trials of an example subject.

We refer the reader to the above analysis in Paragraph 3 of Section 1.4 of the Manuscript:

> "In order to approximate θ and ρ that maximize this likelihood, we performed random search in the space of θ for 1000 iterations (for the convergence of the random search procedure, see Figure S7)."

In addition, it was not clear to me how you dealt with the held out data. Did you fit the model up to the time of the held-out data and then modeled the held out data to check the posterior predictive value? Or was the model fit on the entire data/session and then tested on the held-out data? Did you restart the sequence for learning after the held-out data, or how did you include the sequence dependencies across the boarder of the held out data?

As the Reviewer suggests, we needed to make sure to account for the sequence dependencies between before and after the held-out data in the middle of the sessions, without contaminating the fit with the held-out data. To achieve this, we parsed the entire session in an interrupted manner, updated the ddHCRP with each observation, and computed the model predictions for the response times. This way, all the predicted response times were contingent on all of the participants' previous observations. However, when computing the training log likelihood of the participants' responses, we only took into account the trials that were part of the training set. Therefore, all predictions reflected all previous observations and only predictions on training data were used to optimise the hyperparameters. We highlighted this in the last paragraph of Section 1.4 of the Manuscript:

> "The HCRP parsed the entire e in order to contain sequence knowledge that could explain τ on later segments. But the middle segment of τ was not used for computing the posterior probabilities of θ and ρ. Therefore, all predictions reflected all previous observations and only predictions of the training data were used to optimise the hyperparameters."

Discussion: While you elaborate in the discussion on possible applications e.g. in sequence learning in song birds, I was missing a discussion on how you think the fHCRP in combination with ABC could be implemented in the brain. It would be interesting to read your thoughts on this as a generative model of behavior should neuronally implemented as well.

As the reviewer might imagine, we consider the HCRP to be a computational-level model of chunking. We have added Paragraph 12 to the Discussion in the Manuscript to discuss the question of neural implementation:

> We treat the ddHCRP as a computational-level description of multi-order sequence statistical learning and use, rather than as a process model that could be transparently implementable in neural hardware. Non-parametric Bayesian methods in this family are quite prevalent as computational-level models in cognitive science (e.g., the work of Gershman and Niv on extinction in classical conditioning; and Wolpert and colleagues in motor control), mostly also without suggested neural implementations. However, there has been at least one interesting attempt to link Chinese restaurant processes to

cortico-striatal interactions by Collins and Frank (2013). They developed a cognitive model of structure learning based on the CRP, akin to our model, and a neurobiologically explicit network model as well. Later, they demonstrated that EEG signals were predictive of participants' CRP-like clustering behavior (Collins & Frank, 2016); along with fMRI-based investigations about prefrontal cortical regions involved in cluster creation and use (Donoso et al., 2014). One could perhaps imagine that the rich complexities of the expansive and contractive connections between the cortex, the basal ganglia and the striatum could implement some form of the hierarchy in the ddCRP. Alternatively, purely cortical mechanisms might be involved.

For the case of the bird song, we see the HCRP (the distance dependence is moot in this case), more as a way of determining the depth of context that influences a current syllable - rather than as a model of the way that the bird learns song from his father.

Finally, the ABC fitting is a tool for us, as experimenters, to determine the form of the structure (in this model class) that underpins the behaviour of our participants. We are not in a good position to speculate about the meta-learning that the subjects engage in themselves (i.e., their learning rules for the alpha and lambda parameters) - since the session-by-session changes are modest, and the data are very few.

Availability of data and model: While you say that the relevant data will be made available, it is not clear whether this includes the raw data and the full analysis/modeling code. You have acquired a unique data set which could serve other groups as a basis for their modeling. Please mention the availability of the data within the manuscipt.

As suggested, both the raw data and the full modeling and analysis code has now been made available in a repository: https://github.com/noemielteto/HCRP_sequence_learning, linked in the Data and code availability section of the Manuscript. Over the course of this review process, the raw data were published by Török et al. (2022). We apologise for not publishing the code earlier.

Minor:

Figure 1: The 95% CI are not visible. Are these confidence intervals of the mean. Could show standard deviations instead to increase visibility.

The 95% CI on Figure one are indeed the confidence intervals of the mean and they are barely visible due to their narrowness. In Figure R9, we show the same figure with standard deviation for comparison. However, in the Manuscript, we keep the 95% CI version for coherence, as this notion of error was used in the other figures as well.

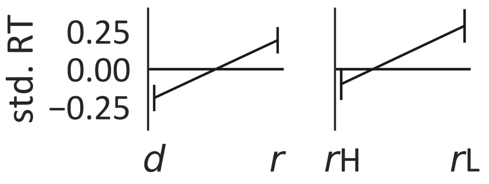

Figure R9. Figure corresponding to Figure 1e of the Manuscript, the error bars indicating the standard deviation instead of the 95% CI.

Figure 5: It was not clear to me whether you applied any correction for multiple comparison for the multiple WM tests and levels of alpha and lambda? Given that you suggest that the detailed model is necessary to extract parameters that relate implicit sequence learning with WM performance, it would be good to state this clearly. In particular, because other studies have suggested there is no relation as you discuss.

In the Manuscript, we present the correlations between the inferred hyperparameters and WM tests that were not corrected for multiple comparisons. We have added this clarification to the Paragraph 2 of Section 2.3 of the Manuscript:

> In case we control for all 24 comparisons presented in the paper, the two significant correlations do not survive the Benjamini&Hochberg correction (their adjusted *p* values increase to .09 and .28, respectively). However, we note that this correction is too harsh, as we did not have prior hypotheses about all of these 24 relationships. In fact, it was expected that the hyperparameters governing the role of very short contexts is not related to WM, as the 1-2 item WM capacity variance is expected to be extremely low in healthy adults. However, we included all comparisons for completeness.

Figure 6c: It would be interesting to discuss not only the trace of sequence learning but also others. Repetition, for example, shows an increase for the last two sessions. What ist the interpretation of this? It is also not clear, what exactly the plotted curves for individual regressors show? Unique variance?

Indeed, the plotted curves in Figure 6c of the Manuscript show the unique variance explained by the model components - we have now made that clear in the caption. As observed by the Reviewer, sequence interference not only reduces the variance explained by the internal sequence model which is full of information about the old sequence, but it also increases the variance explained by the response repetitions and the spatial distance between the current and previous cue. However, at the same time, the coefficients of the low-level effects do not increase – in fact, they mildly decrease as a consequence of the interference, as shown in Figure 4a of the Manuscript (copied here as Figure R10). Therefore, our interpretation is that participants do not ‘fall back’ to rely on aspects of the data other than chunk statistics (in which case our labeling of these effects as ‘low-level’ might be brought into question), but rather that the low-level effects are less obscured by the effects of learning that become obsolete during

interference.

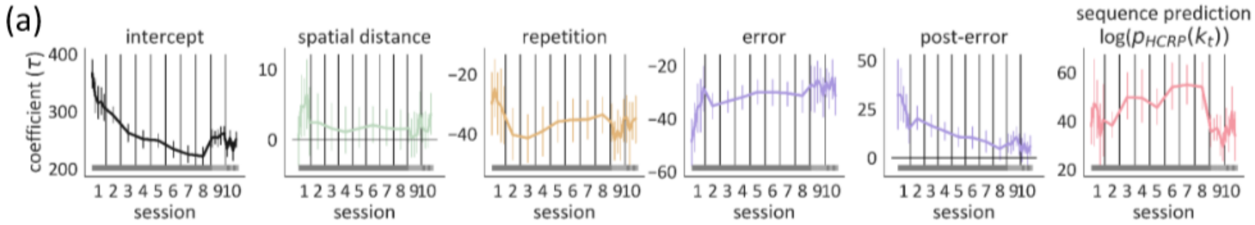

Figure R10. Fitted values of the response parameters in units of τ [ms] (copy of Figure 4a of the Manuscript).

We now make the above point in the Paragraph 6 of the Discussion:

> "Our model was able to account for the initial resistance to interference and slow relearning when a new sequence was introduced in session 9. Sequence interference not only reduced the response time variance explained by the internal sequence model (which was full of information about the old sequence), but also increased the variance explained by low-level effects, such as the response repetitions and the spatial distance between the current and previous cue. However, note that the low-level effect sizes did not increase – in fact, they mildly decreased as a consequence of the interference, as shown in Figure 4a. Therefore, our interpretation is that participants did not 'fall back' to rely on aspects of the data other than chunk statistics (in which case our labeling of these effects as 'low-level' might be brought into question), but rather that the low-level effects were less obscured by the effects of learning that became obsolete during interference."

Priors: When fitting the model with the forgetful prior (Figure 8). Was the posterior value of lambda always at the lowest end of the uniform prior? If so, why did you chose exactly that value?

The Reviewer is quite correct that the posterior value of λ on the trigram level tended to be close to the lowest end of the uniform prior (the value of .0125) (Figure R11, now included as Supplementary Figure S4). To explain: the intent of this part of the paper was to show that it was, in principle, possible to use the ddHCRP to capture aspects of the way that participants commit their errors. However, as noted in the Manuscript, there were insufficient errors (10%) for them to exert sufficient impact on the parameters. Thus, we had to adjust the minimum value of lambda in the prior by hand to force sufficient forgetfulness to show how errors might arise. Whilst acknowledging the artificiality of this procedure, we hope that the result is relevant for future studies where the effects are more pronounced in the erroneous responses, such as explicit sequence prediction tasks.

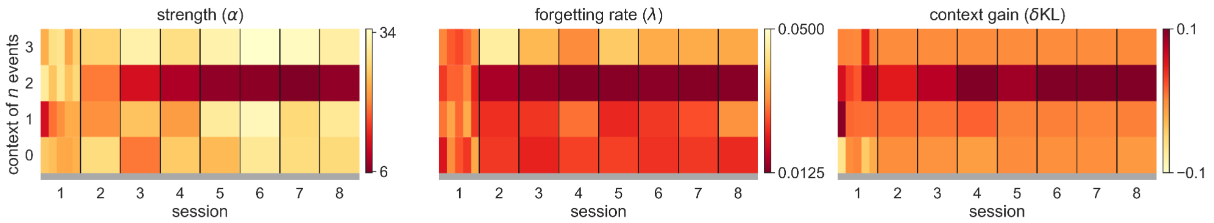

Figure R11. Fitted values of the strength α (left) and forgetting rate λ (middle) parameters are shown, as well as their joint effect on prediction (right), using the constrained prior that places the model in a forgetful regime, described in Table S1. A context of n previous events corresponds to level n in the HCRP. Lower values of α and λ imply a greater contribution from the context to the prediction of behavior. The context gain for context length n is the decrease in the KL divergence between the predictive distribution of the complete model and a partial model upon considering n previous elements, compared to considering only n-1 previous elements. Note that the scale of the context gain is reversed and higher values signify more gain.

We provide this discussion in the Paragraph 7 of the Discussion:

> "Finally, our model class could reproduce error speeding that was specific to the type of errors, that is, whether they reflected the global statistics, local statistics, or no apparent statistics of the task. However, since there were insufficient errors (~10%) in our data for them to exert sufficient impact on the parameters, to examine a ddHCRP account of go wrong more precisely, we had to force the model into a more forgetful regime by adjusting the minimum forgetting rate to a lower level in the prior. Whilst acknowledging the artificiality of this procedure, we hope that the result is relevant for cases such as explicit sequence prediction tasks in which erroneous responses are more prevalent."

Where were the gaussian priors in sessions 2-10 truncated? At the border of the uniform prior in session 1? This was not clear to me.

That is correct – the Gaussian priors in sessions 2-10 were truncated at the border of the uniform prior in session 1. This way we ensured a consistent boundary of the parameter space. We have clarified this in the penultimate paragraph of Section 1.4 of the Manuscript:

> "For the hyperparameters θ, we used a Gaussian prior with a mean of the MAP values of the hyperparameters from the previous bin and a fixed variance, truncated at the boundaries of the uniform prior for session 1 (Table S1, right)."

References

Creel, S. C., Newport, E. L., & Aslin, R. N. (2004). Distant melodies: statistical learning of nonadjacent dependencies in tone sequences. *Journal of Experimental Psychology: Learning, memory, and cognition*, *30*(5), 1119.

Collins, A. G., & Frank, M. J. (2013). Cognitive control over learning: creating, clustering, and generalizing task-set structure. *Psychological review*, *120*(1), 190.

Collins, A. G. E., & Frank, M. J. (2016). Neural signature of hierarchically structured expectations predicts clustering and transfer of rule sets in reinforcement learning. *Cognition*, *152*, 160-169.

Gomez, R. L. (2002). Variability and detection of invariant structure. *Psychological Science*, *13*(5), 431-436.

Romberg, A. R., & Saffran, J. R. (2013). All together now: Concurrent learning of multiple structures in an artificial language. *Cognitive science*, *37*(7), 1290-1320.

Szegedi-Hallgató, E., Janacsek, K., Vékony, T., Tasi, L. A., Kerepes, L., Hompoth, E. A., ... & Németh, D. (2017). Explicit instructions and consolidation promote rewiring of automatic behaviors in the human mind. *Scientific Reports*, *7*(1), 1-7.

Török, B., Nagy, D. G., Kiss, M., Janacsek, K., Németh, D., & Orbán, G. (2022). Tracking the contribution of inductive bias to individualised internal models. *PLOS Computational Biology*, *18*(6), e1010182.

Van den Bos, E., Christiansen, M. H., & Misyak, J. B. (2012). Statistical learning of probabilistic nonadjacent dependencies by multiple-cue integration. *Journal of Memory and Language*, *67*(4), 507-520.
