# Supplementary material for: Tracking human skill learning with a hierarchical Bayesian sequence model
Source: PLoS Comput Biol. 2022 Nov 30;18(11):e1009866. doi: 10.1371/journal.pcbi.1009866 (PMC9744313; doi:10.1371/journal.pcbi.1009866)
Supplement: Attachment — Submitted filename: response_letter_2.pdf [file pcbi.1009866.s010.pdf]

Dear Doctors Mathys and Gershman

Please find enclosed the revised version of our previous submission entitled “Tracking human skill learning with a hierarchical Bayesian sequence model”. We thank you and the reviewers for giving further feedback on our manuscript. We were delighted that Reviewer #1 found that the previously raised concerns were fully addressed and our manuscript can make a contribution to the field in its state after the first revision. In this second revision, we have carefully addressed the remaining comments by Reviewer #2 and Reviewer #3. Moreover, we made slight adjustments to the Manuscript to improve clarity, in the first paragraph of the abstract and the first paragraph of the discussion.

We provide a narrative response to the reviewers’ concerns below. The reviewers’ comments are color-coded black and the authors’ comments are green. Our modifications in the Manuscript are highlighted in red. The modified segments of the Manuscript that were copied in the response letter are indented. The copied segments are identical between the Manuscript and the responses except for the numbering and referencing of the figures, for the sake of readability.

Sincerely,

Noémi Éltető, Dezső Németh, Karolina Janacsek, Peter Dayan

Reviewer #2: The authors answered all my concerns. I appreciate in particular the additional model comparison and algorithm explanation. I still found the provided code a bit lacking (I expected a README and comments structuring the code: the repo is so far quite bare and unusable without some serious guessing work). I trust the authors to address this last point before publication. I otherwise have no further comments.

We apologise that our repository and, in particular, the code documentation, was a bit basic. The reason for this was that we expected further potential changes to the code and we planned to wait with the detailed documentation until the code was finalised. We now have included a README, as well as documentation for each function in the ddHCRP library. We believe that this ensures the reproducibility of our results, as well as the usability of our HCRP sequence model implementation for future projects.

Reviewer #3: Comments on Revised manuscript "Tracking human skill learning with a hierarchical Bayesian sequence model".

In this revision, the authors have addressed my concerns in a satisfactory manner. I have two comments remaining directly related to their answers.

Jakob Heinzle

Major:

I was a bit surprised about your statement that the data was already published in the recently accepted paper by Török et al. (PLoS Comput Biol 18(6):e1010182. https://doi.org/10.1371/journal.pcbi.1010182). I understand this is exactly the same data that you analyse, here. I had not realized this when reading the initial manuscript, and I think it should be made more transparent. While you cited the Török study (a preprint version of it) for additional reference on the methods, I could not find a statement that it is indeed the same data that you are using. I consider it as fundamentally important to mention in your paper that it is not the first time this data are published. Readers need to understand that you present a novel modeling analysis, but of an existing data set. Also, in the discussion, you should mention that the results of Török et al rest on exactly the same data. This is important for the questions regarding model comparison that other reviewers have brought up and which I think are highly relevant. Note: I added this point as major, not because I think that it will entail a lot of work, but because of its importance.

We agree with the importance of transparency, and we therefore further highlighted that we are modeling the same data as Török et. al. (2022). We have done this in Paragraph 1 of the Methods section:

“A detailed description of the task, procedure, and participants can be found in \cite{torok2022tracking} where this data was first published.”

as well as in Paragraph 9 of the Discussion:

“The other, contemporaneous alternative account \cite{torok2022tracking} is mechanistic, and so is rather closer to ours. \cite{torok2022tracking} fitted their model to the same data set that we present here, therefore the differences between our models and fitting procedures are straightforwardly understood and we will elaborate on them here. They use an infinite hidden Markov model (iHMM) [...]”

We have also pointed out two other relevant differences between our modeling approaches in Paragraphs 9 and 10 of the Discussion:

“Our model, apart from capturing parsimony, also captured the nonstationarity of humans' expectations. Thus, it explained higher-order perseveration effects whereby recent chunks had a greater expectation of reccurence.”

“Second, instead of treating the learning sessions independently, we assumed that participants refine the same internal model with new information session by session, as well as by adjusting the hyperparameter set of that model (i.e. *how* the learned information used).”

During this revision process, the Török study was published here in PLoS CB, so we have updated our reference to this peer-reviewed version of their manuscript.

Minor:

There are still some things unclear about ABC. E.g. what stopping criterium do you use.

Thank you for pointing out the lack of clarity here. We did not set a stopping criterion, rather, we ran the search for 1000 iterations in the case of each data bin (epoch or session) and each participant irrespectively of convergence. We repeated the search procedure 10 times in order to produce 10 samples from the posterior distribution. We apologise that this detail was missing; we have now added it in Paragraph 3 of the Methods section:

“We repeated the search procedure 10 times, yielding 10 samples from the posterior distribution, and Θ associated with the highest likelihood of participants' responses was chosen as the MAP estimate of the hypermarameters.”

[...] also, it is clear that using 5 instantiations of the CRP is not reaching a plateau. Why is 5 still a good number?

Although it is true that 5 CRP samples don't reach the plateau, our analyses on an example data segment, showed that the median variation of predictions was only 3%. The computational cost of fitting is rather large: we update the sequence model trial by trial, and we fit the model session by session, conditioning later sessions on earlier ones, for a total of 20825 trials per participant and 25 participants in total. Thus, increasing the number of CRP samples to 10 (which would have been the plateau) would have meant several days of additional computation - for a reduction in variation that we suggest would not have enriched our results in a meaningful way.

In addition, I think you need to mention more clearly that your held out dataset is not fully independent. I understand that you need to include the trials of the middle segment (the held out data) in your CRP updates to give the model the right context for the later trials. However, this means that your parameter estimates are conditional on the inputs ("features") of the training set, even if you do not use the RT measurement of that period to fit the hyperparameters. In machine learning, one would usually not include the features of the test set in the training, even if test labels are not used. I realize that this is a tricky point and, hence, I think it is important that you explain this deviation from an ideal held-out set to the reader.

We agree that our method needs to be clearly distinguished from the simplest form of testing on held-out sets. It is important to note that the feature of the data that is not held out is the stimuli with which the participants were presented, whereas their responses were completely held out. While not conventional in machine learning, this is often the 'ideal' approach in cognitive science. In our case, if we removed the middle segment of a session completely, that corresponds to assuming that the participant is behaving as if she has learnt nothing during the middle of the session. This would lead to hyperparameter estimates reflecting better memory, in order to compensate for the relative lack of observations, which was undesirable. We emphasized the necessary divergence from the conventional machine learning method in Paragraph 5 of the 1.4 Parameter fitting subsection:

"Holding out the responses but not the observations, instead of completely held-out data as typical in machine learning, was essential to provide the model with the right context for predicting behavior, whilst ensuring that it was not contaminated with test behavior."
